# Festigkeit der Konusverbindung modularer Revisionshüftschäfte: Einfluss der Verunreinigung der Konusverbindung und der Fügebedingung auf die Relativbewegung und Abzugskraft zwischen Schaft und Halsteil der MRP-Prothese

**DOI:** 10.1007/s00132-023-04459-2

**Published:** 2023-12-14

**Authors:** Julius M. Boettcher, Kay Sellenschloh, Anna Strube, Gerd Huber, Michael M. Morlock

**Affiliations:** Institut für Biomechanik, Technische Universität Hamburg, Denickestraße 15, 21073 Hamburg, Deutschland

**Keywords:** Korrosion, Gelenkerkrankungen, Revisionschirurgie, Implantatbruch, Totaler Hüftgelenkersatz, Corrosion, Joint diseases, Revision surgery, Implant fracture, Total hip arthroplasty

## Abstract

**Einführung:**

In der Revisionschirurgie ermöglichen modulare Implantatkomponenten dem Chirurgen, die Eigenschaften des Implantats an die Knochensituation anzupassen. An der modularen Verbindung kann es zu Relativbewegungen kommen, die zu Abrieb und nachfolgenden biologischen Reaktionen führen, insbesondere aufgrund einer unvollständigen Montage und Kontamination der konischen Verbindung. Ziel dieser Studie war es, zu zeigen, ob eine unvollständige Montage und eine versehentliche Kontamination des modularen Konus eine Veränderung der Verbindungsstärke verursacht.

**Material und Methoden:**

Modulare Konusverbindungen zwischen Hals und Schaft (*n* = 48) wurden in sieben Gruppen eingeteilt, die sich hinsichtlich des Verunreinigungsgrades (nativ, kontaminiert, gereinigt) und der Fügebedingungen (gesichert, vorgespannt und gesichert) unterschieden, und nach dem Fügen mit einer servohydraulischen Prüfmaschine zyklisch belastet wurden. Die Kontamination wurde durch eine Kombination aus Schweineknochenpartikeln und Rinderblut erreicht. Für jede Gruppe wurde zusätzlich die Anzahl der Umdrehungen des Drehmomentbegrenzers beim Sichern der Konusverbindung erfasst. Mittels digitaler Bildkorrelation wurden die Verdrehung des Halsteils, die Mikrobewegung und das axiale Setzen des Halsteils ermittelt. Anschließend wurden die Abzugskräfte als Maß für die verbleibende Verbindungsfestigkeit der Konusverbindung bestimmt.

**Ergebnisse:**

Eine Verunreinigung der Konusverbindung, insbesondere in Kombination mit einer unsachgemäßen Montage der Komponenten, erhöhte signifikant die Rotation (35,3 ± 13,7° vs. 2,4 ± 4,4°; p <0,001), die Mikrobewegung (67,8 ± 16,9 μm vs. 5,1 ± 12,1 μm, p <0,001) und das axiale Setzen (‑34,1 ± 16,9 μm vs. 4,3 ± 10,9 μm; p <0,001) des Halses gegenüber dem Schaft.

**Schlussfolgerung:**

Intraoperativ lässt sich eine Kontamination der Konusoberfläche daran erkennen, dass beim Festziehen der Sicherungsschraube mehrere Umdrehungen erforderlich sind. Eine korrekte Reinigung mit dem neuen Konusreinigungsinstrument und eine vollständige Montage mit Vorspannen der Komponenten können das Risiko eines frühzeitigen Versagens und eines Ermüdungsbruches der modularen Konusverbindung verringern.

## Abstract (en)

**Introduction:**

In revision surgery, modular implant components allow the surgeon to tailor the characteristics of the implant to the bone situation. Relative motion can occur at the tapered modular connection, leading to fretting corrosion and subsequent biological reactions, particularly due to poor assembly and contamination of the tapered connection. The aim of this study was to demonstrate whether incomplete assembly and inadvertent contamination of the modular taper causes a change in junction strength.

**Material and methods:**

Modular taper junctions between the neck and the stem (*n* = 48) were divided into seven groups that differed with respect to contamination (native, contaminated, cleaned) and assembly conditions (secured, pre-tensioned and secured). Contamination was achieved by a combination of porcine bone particles and bovine blood. For each group, the number of rotations of the torque limiter while securing the conical connection was recorded. The implants were subjected to cyclic loading. DIC was used to determine neck rotation, micromotion and axial subsidence. Loosening torque of the locking screw and pull-off forces were measured as an equivalent of residual taper junction strength.

**Results:**

Contamination of the taper junction, especially in combination with improper assembly of the components, significantly increased the rotation (35.3 ± 13.7° vs. 2.4 ± 4.4°; p <0.001), micromotion (67.8 ± 16.9 μm vs. 5.1 ± 12.1 μm, p <0.001) and axial subsidence (‑34.1 ± 16.9 μm vs. 4.3 ± 10.9 μm; p <0.001) of the neck relative to the stem.

**Conclusion:**

Intra-operatively, contamination of the taper surface can be identified by the need for multiple turns when tightening the locking screw. Correct cleaning with the new taper cleaning instrument and complete assembly with pre-tensioning may reduce the risk of early failure and fatigue fracture of the modular taper connection.

**Graphic abstract:**

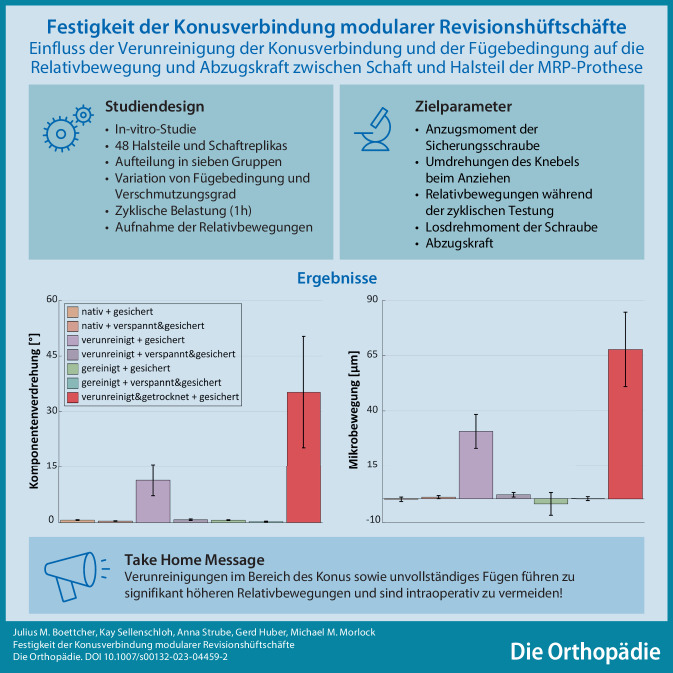

## Hinführung zum Thema

Modulare Revisionsendoprothesen für die Hüfte bieten für Patienten mit proximalen Knochendefekten und einem insgesamt reduzierten Knochenbestand durch die intraoperative Anpassungsfähigkeit an die individuelle Patientenanatomie Vorteile gegenüber Monoblockimplantaten [3, 7]. Die Fügestellen der Komponenten einer modularen Prothese stellen durch die Durchmesserverkleinerung jedoch eine entscheidende Schnittstelle im Kontext der mechanischen Dauerfestigkeit des Implantats dar, weshalb eine korrekte intraoperative Fügung von entscheidender Bedeutung ist. Die Auswirkungen einer unsachgemäßen Fügung sowie der Einfluss der Verunreinigung der Oberflächen durch Körperflüssigkeiten und Partikel (z. B. Knochenreste) auf die Verbindungsfestigkeit der Konusverbindung sind daher von ausschlaggebender Bedeutung für die Langlebigkeit der Versorgung, um Korrosion an der Schnittstelle bzw. einen Konusbruch zu vermeiden.

## Einleitung

Die Implantation einer Hüfttotalendoprothese ist ein weit verbreitetes und überaus erfolgreiches chirurgisches Verfahren zur Schmerzlinderung und Wiederherstellung der Gelenkfunktionalität bei Patienten mit Hüftgelenkserkrankungen [14]. Revisionsoperationen an der Hüfte, bei denen die Implantate ersetzt werden müssen, stellen aufgrund des häufig reduzierten femoralen Knochenangebots eine besondere Herausforderung dar [1].

Modulare Revisionsprothesen bieten dem Operateur die Flexibilität, die Konfiguration der Implantatkomponenten intraoperativ an die individuelle Patientensituation anzupassen, um die anatomische Situation speziell bei Revisionseingriffen bestmöglich zu rekonstruieren [3, 7]. Die dynamischen Kräfte und Momente, die bei Belastungsspitzen, wie beispielsweise Stolpern oder Stürzen, insbesondere bei übergewichtigen Patienten, von der Prothese übertragen werden müssen, können jedoch die Belastungsgrenze des Implantats überschreiten, insbesondere an den Verbindungsstellen, da hier der Durchmesser deutlich unter dem übrigen Implantatdurchmesser liegt [8].

Auch ohne unmittelbaren Implantatbruch zur Folge zu haben, kann diese Überbelastung zu Relativbewegungen führen, die wiederum zu Korrosion und in der Folge zu biologischen Reaktionen durch abriebbedingte Freisetzung von Metallionen führen können [15]. Durch diesen Effekt steigt das Risiko von Komplikationen wie Nekrose, Entzündung, Osteolyse und aseptischer Lockerung [2, 11]. Darüber hinaus kann ein Materialverlust am Schaftkonus zu einer Lockerung der Konusverbindung führen [12].

Verunreinigungen der Verbindung, insbesondere intraoperative Rückstände aus Blut oder Knochenpartikel, oder unzureichende Fügung haben sich als wesentlicher Risikofaktor für diese Art von Versagen erwiesen [6, 13]. Ziel dieser Studie war es daher, den Einfluss von Verunreinigung (inkl. der Reinigung zuvor verunreinigter Konusverbindung mit einem entsprechenden Instrument) sowie unvollständiger Fügung auf die Stabilität der Konusverbindung modularer Revisionshüftschäfte zu untersuchen.

Es wurde erwartet, dass die Verbindungsfestigkeit sowohl bei verunreinigten Konen als auch bei nicht korrekt gefügten, sondern nur gesicherten Verbindungen, signifikant reduziert ist.

## Material und Methoden

**Figure 1:**
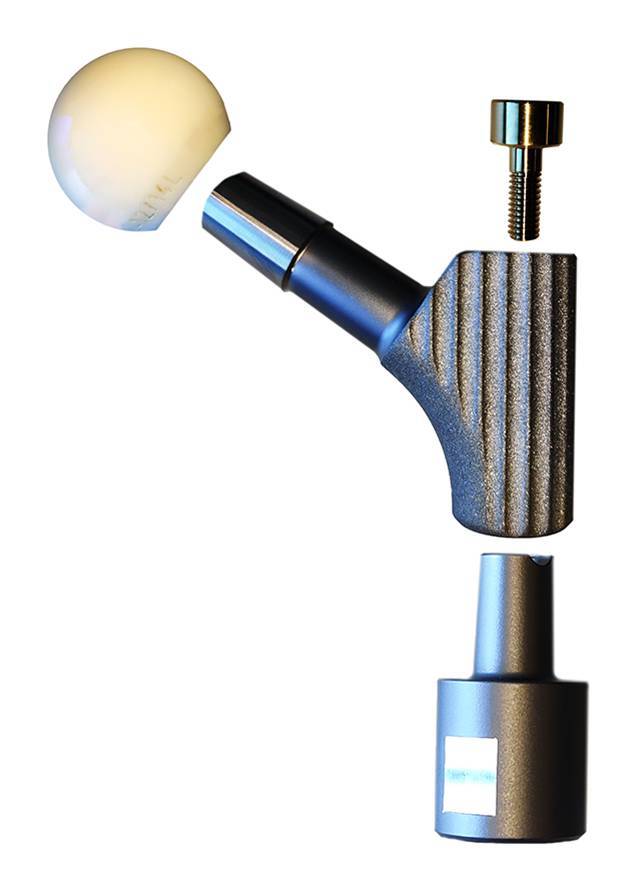

Die Untersuchung wurde an der Schaft-Halsstück-Konusverbindung eines gängigen Revisionssystems an Originalkomponenten durchgeführt (MRP-TITAN®, PETER BREHM GmbH, Weisendorf, Deutschland). Um den „worst case“ zu simulieren, wurden kurze Halsstücke („S“ lateralisiert, Offset 50 mm [8]) mit Keramikköpfen (28 mm, 12/14L) verwendet. Anstelle von vollständigen Schäften wurden vom Hersteller gekürzte Schaftkonusreplika für die Testung zur Verfügung gestellt, welche mit demselben Fertigungsablauf wie reguläre Schäfte gefertigt wurden, sich aber besser zur Montage in der Materialprüfmaschine eignen (Abb. 1). Der Konuswinkel des männlichen Konus des modularen Schaftes und des weiblichen Konus des Prothesenhalses wurden mithilfe eines CNC-gesteuerten Koordinatenmessgerätes mit einer Tastkugel aus Siliziumnitrid (Ø 2 mm) vermessen und die absolute Winkeldifferenz ermittelt (CRYSTA-Apex S574, Mitutoyo Deutschland GmbH, Neuss, Deutschland; Kalibriergenauigkeit < 3 μm; 50 Messpunkte pro Konus).

Der Einfluss der Verunreinigung der Konusverbindung auf die Fügefestigkeit wurde für drei Zustände untersucht: „nativ“, „verunreinigt“ sowie „verunreinigt und gereinigt“, der Einfluss der Fügung wurde anhand der Fügezustände „verspannt und gesichert“ (entsprechend der Instrumentationsanleitung [16]) sowie „nur gesichert“ untersucht. Jede Kombination wurde sieben Mal getestet (Gesamtanzahl der Testungen 3 × 2 × 7 = 42). Die numerische Aufbereitung sämtlicher Messdaten erfolgte mittels Matlab (Version R2022b, The Mathworks, Natick, MA, USA). Die Fügung gemäß Gebrauchsanleitung erfolgte durch Verspannen mittels des torsionsfreien Vorspanninstrumentes (TOV), gefolgt von der Sicherung mit einer Sicherungsschraube. Für die Bedingung „nur gesichert“ erfolgte keine Verspannung. Für beide Bedingungen betrug das unter Verwendung des Drehmomentbegrenzers aufgebrachte Anzugsmoment der Sicherungsschraube 25 Nm (gemäß Instrumentationsanleitung [16]). Die mittels des TOV aufgebrachte Vorspannkraft beträgt laut Herstellerangaben 11 kN und wird durch den Einsatz eines Abreißbolzens gewährleistet. Die Prothesenkomponenten wurden vor der Vermessung auf die sechs Gruppen aufgeteilt. Als „Worst-case“-Konfiguration wurden zusätzlich sechs Konusverbindungen „verunreinigt und getrocknet“ in Verbindung mit der Fügebedingung „nur gesichert“ untersucht. Hierbei wurde zwischen dem Zeitpunkt der Verunreinigung und dem Fügen eine Stunde gewartet, bis das biologische Material vollständig koaguliert war.

**Figure 2:**
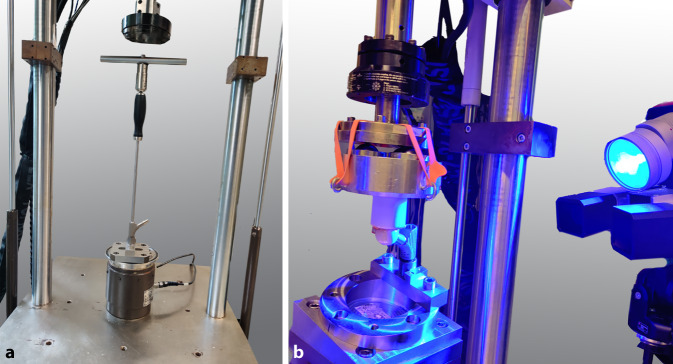

Das maximale Anzugsmoment der Sicherungsschraube vor Auslösen des Drehmomentbegrenzers wurde mit der Kraftzelle einer servohydraulischen Materialprüfmaschine aufgezeichnet (MiniBionix II, Lastgrenzen der Kraftzelle 25 kN, 250 Nm, MTS, Eden Prairie, MN, USA, Abb. 2a). Die Anzahl der notwendigen Umgriffe beim Drehen des Knebels bis zum Auslösen des Drehmomentbegrenzers wurde hierbei bestimmt, um den Setzweg des Halsstückes während des Anziehens der Sicherungsschraube bestimmen zu können.

Nach dem Fügevorgang wurden alle Prüfkörper mit einer separaten servohydraulischen Prüfmaschine (Bionix 858, Lastgrenzen der Kraftzelle 10 kN, 100 Nm, MTS) in Anlehnung an die Prüfparameter der Norm ASTM F1875 [5] zyklisch, mit konstanter Ober- und Unterlast belastet (Methode 2b, sinusförmiger Lastverlauf, 1 Hz, 50–3000 N, 3600 Zyklen). Die Prüfkörper wurden entsprechend der Prüfanordnung zur Bestimmung der Dauerwechselfestigkeit nach ISO 7206‑4 [10] ausgerichtet (10° laterale Neigung, 9° dorsale Neigung, Abb. 2b).

**Figure 3:**
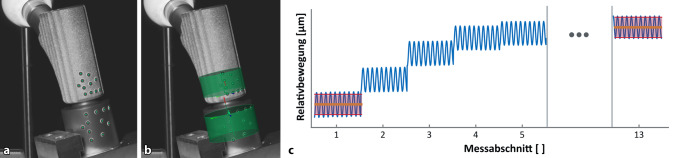

Während der Belastung wurde die Relativbewegung an der Konusverbindung zwischen den beiden Komponenten mit einem digitalen Bildkorrelationssystem (DIC) alle 300 s für 10 s (12 Messintervalle) sowie initial 30 s nach Start der Testung berührungslos erfasst (Aramis 3D, MV 100, GOM, Braunschweig, Deutschland; Auflösung 2752 × 2200 Pixel, Messgenauigkeit 0,01 Pixel [Optimierter Kalibrierfehler analog zu [17]], Messvolumen: 100 × 80 × 50 mm^3^, Bildwiederholungsrate: 25 Hz). Hierfür wurden kreisförmige Klebemarkierungen (Ø 0,4 mm) auf der lateralen Seite der beiden Implantatkomponenten angebracht. Die Relativbewegungen wurden mittels der Software GOM Correlate analysiert (Version 2018, GOM, Braunschweig, Deutschland). Die 3‑D-Koordinaten der beiden Komponenten wurden digital anhand von Zylindern bestimmt, die an die Marker des Halsstückes und des Schaftes eingepasst wurden (Abb. 3a,b). Die Relativbewegung des Halsteils in Richtung der Achse des Schaftes sowie die Verdrehung der Komponenten um die Schaftachse wurden hieraus bestimmt. Als Setzen wurde die Änderung des Mittelwerts der axialen Relativbewegung zwischen dem ersten und dem letzten Messabschnitt definiert, als Mikrobewegung die Änderung der Standardabweichung der axialen Relativbewegung ebenfalls zwischen dem ersten und dem letzten Messintervall (Abb. 3c). Die Standardabweichung wurde aus Gründen der Robustheit der Auswertung der absoluten Amplitude vorgezogen. Bei einer idealen harmonischen Schwingung entspricht die Standardabweichung etwa 70 % der Amplitude. Ein negativer Wert der Mikrobewegung entspricht einer Verringerung der Relativbewegung im Verlauf der zyklischen Belastung. Die relative Verdrehung des Halsstückes auf dem Schaft um die Schaftachse wurde als 2‑Richtungs-Winkel zwischen den Lotlinien der beiden Zylinder zwischen erstem und letztem Messintervall bestimmt.

Im Anschluss an die zyklische Belastung wurde das Losdrehmoment der Sicherungsschraube sowie die Abzugskraft der Konusverbindung mit der Kraftzelle der zur Belastung benutzten Prüfmaschine bestimmt (Lastgrenzen 25 kN, 250 Nm).

Die proximale Implantatkomponente wurde mit einer Abzugsgeschwindigkeit von 0,008 mm/s gemäß ISO 7206-10 [9] und ASTM F2009 [4] abgezogen und die maximale Abzugskraft bestimmt.

### Verunreinigung der Konusverbindung (Schaft/Halsstück)

**Figure 4:**
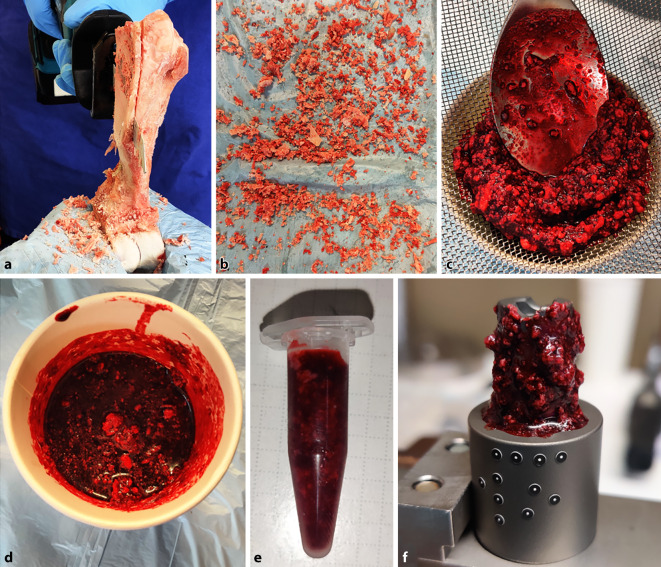

Für die Verunreinigung der Konusverbindung wurden mittels einer elektrischen Säbelsäge (DJR183Z, Makita, Anjo, Japan) mit eingesetztem Säbelsägeblatt mit Zahnung für Holz und Metall (S 922 HF, Bosch, Gerlingen, Deutschland) Partikel aus porzinen Oberschenkelknochen (Lebensmittelqualität, gefroren gelagert bei −22 °C) generiert. Die erzeugten Partikel wurden anschließend mit bovinem Blut (Lebensmittelqualität, frisch, ohne Zusatz von Antikoagulanzien) gravimetrisch zu gleichen Teilen gemischt und gesiebt (Maschenweite 1,6 mm), um die maximale Partikelgröße zu begrenzen. Die erhaltene Suspension wurde abschließend in Portionen von 1,5 ml auf Reaktionsgefäße (SafeSeal, Sarstedt, Nümbrecht, Deutschland) verteilt und bei −22 °C bis zur Verwendung gelagert, um eine vorzeitige Degeneration auszuschließen (Abb. 4a–e).

Die Blut-Partikel-Suspension wurde 30 min vor der Testung aufgetaut und mit einem abriebfesten Kunststoffspatel in Gänze auf die Konusoberfläche aufgetragen. Dabei wurde das Gewinde ausgespart, da intraoperativ das Eindringen von Partikeln in die Gewindegänge durch den Führungsstab verhindert wird (Abb. 4f).

### Reinigung der Konusverbindung (Schaft/Halsstück)

**Figure 5:**
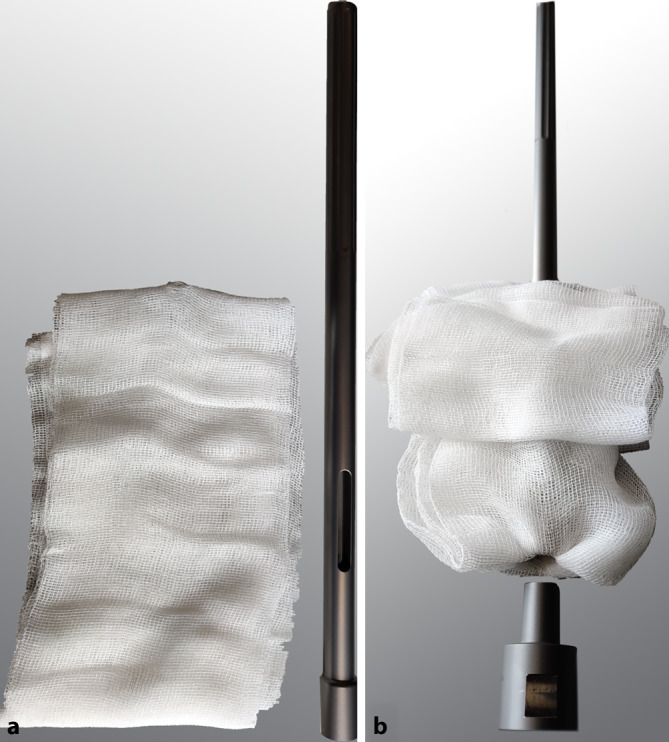

**Figure 6:**
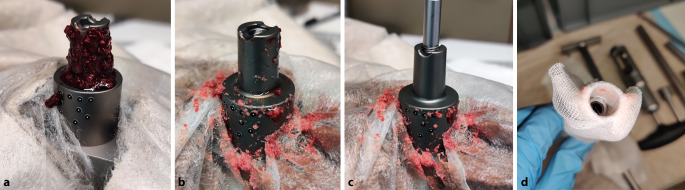

Für die „gereinigten“ Gruppen wurden vor dem Fügen die verunreinigte Konen zunächst mit 10 ml NaCl-Lösung (NaCl 0,9 %, Braun, Melsungen, Deutschland) gespült und anschließend zweimal mit dem neuen Reinigungsinstrument des Implantatherstellers (Safety Wiper MRP-TITAN®, PETER BREHM GmbH, Abb. 5), in welchem Mullbinden (ES-Kompressen 10 cm x 20 cm, Paul Hartmann AG, Heidenheim, Deutschland) eingelegt werden, gereinigt und getrocknet (Abb. 6a–d).

### Statistische Auswertung

Die statistische Auswertung wurde mit SPSS (Version 26, IBM Statistics, Amonk, NY, USA) durchgeführt. Die ermittelten Messwerte wurden auf Normalverteilung und Homoskedastizität geprüft und anschließend parametrische Mittelwertvergleiche (ANOVA) sowie Korrelationsanalysen nach Pearson durchgeführt. Ein Post-Hoc-Test nach Tukey wurde angewendet, um Unterschiede zwischen den Gruppen zu identifizieren. Zudem wurden t‑Tests für einen paarweisen Vergleich der Gruppe „verunreinigt + verspannt und gesichert“ mit der Gruppe „nativ + verspannt und gesichert“ bezüglich der Mikrobewegung, der Komponentenverdrehung sowie des axialen Setzens durchgeführt, um den isolierten Einfluss der Verunreinigung auf die Relativbewegung zu untersuchen Der Typ-I-Fehler (α-Level) wurde auf 5 % gesetzt.

## Ergebnisse

Die mittlere Winkeldifferenz zwischen den Konuswinkeln des weiblichen Konus des Halsteils und des männlichen Konus des Schafts betrug 0,026° (Standardabweichung 0,013°) und wies keinen Unterschied zwischen den Gruppen auf (*p* = 0,824). Somit kann von einem flächigen Kontakt der Konen bei allen Gruppen ausgegangen werden.

Die gemessenen Anzugsmomente lagen innerhalb der Herstellerspezifikationen. Der Anzugmoment der Gruppe „gereinigt + gesichert“ wies dabei ein leicht, aber signifikant höheres Anzugsmoment auf, als alle anderen Konfigurationen (26,2 ± 0,4 Nm vs. 25,4 ± 0,3 Nm; *p* < 0,001; Tab. 1), ansonsten wurden keine Unterschiede zwischen den Gruppen festgestellt.

**Table 1:** 

Gruppe	Anzugsmoment (Nm)	Umgriffe ( )	Mikrobewegung (µm)	Setzen (µm)	Verdrehung (°)	Losdrehmoment (Nm)	Abzugskraft (kN)
1	25,4 ± 0,3	2,0 ± 0,0	−0,3 ± 1,0	1,4 ± 5,1	0,7 ± 0,1	1,1 ± 0,4	8,3 ± 0,5
**2**	**25,3 ± 0,1**	**1,1 ± 0,4**	**0,7 ± 0,7**	**−0,8 ± 1,6**	**0,4 ± 0,1**	**5,2 ± 1,4**	**10,4 ± 0,4**
3	25,6 ± 0,7	4,8 ± 1,0**	30,7 ± 7,7**	−24,4 ± 13,7**	11,4 ± 4,2**	2,9 ± 2,4	8,2 ± 1,0
4	25,4 ± 0,2	1,0 ± 0,0	1,8 ± 1,0*	−0,1 ± 4,5	0,8 ± 0,2*	7,1 ± 4,4	10,9 ± 1,0
5	26,2 ± 0,4	2,1 ± 0,9	−2,4 ± 5,2	−1,7 ± 5,7	0,7 ± 0,1	1,7 ± 0,9	7,7 ± 0,2
6	25,5 ± 0,2	1,0 ± 0,0	0,2 ± 0,9	−0,4 ± 0,5	0,3 ± 0,1	5,9 ± 1,5	10,4 ± 0,2
7	25,3 ± 0,1	8,5 ± 2,4**	67,8 ± 16,9**	−34,1 ± 16,9**	35,3 ± 15,1**	3,0 ± 1,7	6,9 ± 1,6

1 – nativ + gesichert, 2 – nativ + verspannt und gesichert (Referenz), 3 – verunreinigt + gesichert, 4 – verunreinigt + verspannt und gesichert, 5 – gereinigt + gesichert, 6 – gereinigt + verspannt und gesichert, 7 – verunreinigt und getrocknet + gesichert

** signifikant erhöht im Vergleich zu den anderen Gruppen, * signifikant erhöht im Vergleich zur Referenz Gruppe 2

Die Anzahl der notwendigen Umgriffe beim Drehen des Knebels bis zum Auslösen des Drehmomentbegrenzers war für die Gruppen „verunreinigt + gesichert“ (4,8 ± 0,9 Umgriffe) sowie „verunreinigt und getrocknet + gesichert“ (8,5 ± 2,1 Umgriffe) signifikant höher als bei den anderen Gruppen (2,0 ± 1,4 Umgriffe, *p* < 0,001; Tab. 1), die sich nicht voneinander unterschieden.

**Figure 7:**
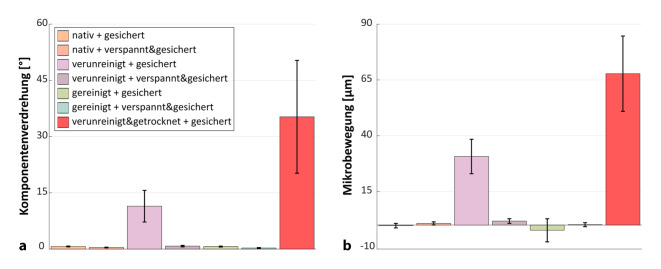

Die Verdrehung des Halsstückes auf dem Schaft als Folge der zyklischen Belastung war für die Gruppen „verunreinigt + gesichert“ (11,43 ± 3,90°) sowie „verunreinigt und getrocknet + gesichert“ (35,28 ± 13,74°) signifikant größer als bei den üblichen Gruppen (2,37 ± 4,36°; *p* < 0,001 für beide; Abb. 7a; Tab. 1). Im direkten paarweisen Vergleich zeigte darüber hinaus die Gruppe „verunreinigt + verspannt und gesichert“ eine signifikant höhere Verdrehung als die Gruppe „nativ + verspannt und gesichert“ (0,8 ± 0,2° vs. 0,4 ± 0,1°, *p* = 0,001, t‑Test).

Die Mikrobewegung in der Konusverbindung war für die Gruppen „verunreinigt und getrocknet + gesichert“ (67,8 ± 16,9 µm) sowie „verunreinigt + gesichert“ (30,7 ± 7,7 µm) signifikant höher als in den anderen Gruppen (5,1 ± 12,1 µm, *p* < 0,001 für beide; Abb. 7b; Tab. 1). Auch für die Mikrobewegung zeigte im direkten paarweisen Vergleich die Gruppe „verunreinigt + verspannt und gesichert“ signifikant höhere Mikrobewegungen als die Gruppe „nativ + verspannt und gesichert“ (1,8 ± 1,0 µm vs. 0,7 ± 0,7 µm, *p* = 0,044, t‑Test).

Für das axiale Setzen als Folge der zyklischen Belastung zeigte sich ein analoges Ergebnis: Die Gruppen „verunreinigt + gesichert“ (−24,4 ± 13,7 µm) und „verunreinigt und getrocknet + gesichert“ (−34,1 ± 16,9 µm) wiesen ein signifikant höheres Setzen im Vergleich mit den übrigen Gruppen auf (−4,3 ± 10,9 µm; *p* < 0,001; Tab. 1). Zwischen den übrigen Gruppen wurden keine Unterschiede festgestellt.

Die Losdrehmomente der Sicherungsschraube nach der zyklischen Belastung waren in den verspannten und gesicherten Gruppen (6,08 ± 2,73 Nm) signifikant höher als in den lediglich gesicherten Gruppen (2,13 ± 1,63 Nm; *p* < 0,001; Tab. 1).

Die Abzugskräfte zum Trennen der Konusverbindung nach der zyklischen Belastung verhielten sich genauso: Die verspannten und gesicherten Gruppen (10,54 ± 0,62 kN) zeigten noch die nahezu aufgebrachte Vorspannkraft von 11 kN auf, die nur gesicherten Gruppen hingegen eine um etwa 30 % reduzierte Abzugskraft (7,80 ± 1,02 kN; *p* < 0,001; Tab. 1). Die verunreinigten und anschließend gereinigten Proben zeigten annähernd identische Abzugskräfte wie die nativen Proben. Die Streuung der Abzugskräfte war bei den verunreinigten Gruppen deutlich höher als bei den nativen und den verunreinigten und anschließend gereinigten Proben (Tab. 1).

## Diskussion

Die Hypothese, dass ohne Vorspannung gefügte Konusverbindungen eine schlechtere Verbindungsfestigkeit aufweisen als vorgespannte Komponenten, wurde eindeutig bestätigt.

Die Hypothese, dass eine Verunreinigung der Konusoberflächen zu einer Abnahme der Verbindungsfestigkeit führt, also Losdrehmoment und Abzugskraft, konnte jedoch nicht bestätigt werden. Allerdings war die Verdrehung des Halsstückes auf dem Schaft und die Mikrobewegung zwischen Halsstück und Schaft signifikant erhöht.

Bei der überwiegenden Zahl der ausgewerteten Parameter zeigte sich jedoch eine größere Streuung bei den verunreinigten und nicht gereinigten Gruppen im Vergleich zu den gereinigten bzw. nativen Gruppen – welche untereinander keine signifikanten Unterschiede aufwiesen. Dies hebt die Bedeutung der Reinigung hervor. Insbesondere bei der für die Dauerfestigkeit entscheidenden Mikrobewegung konnte durch die Reinigung eine signifikante Reduktion im Vergleich zu den nicht gereinigten Gruppen und damit Werte wie im nativen Zustand erreicht werden. Die „Worst-case“-Konfiguration „verunreinigt und getrocknet + gesichert“ zeigte die schlechtesten Werte hinsichtlich aller evaluierten Parameter.

Die Verunreinigung des Konus konnte durch das neue Instrument zur Reinigung der Konusoberfläche reproduzierbar entfernt werden. Es ist zu erwarten, dass durch die Reinigung mit dem neuen Instrument und die damit verbundene signifikante Reduzierung der Mikrobewegung das Risiko von Reibkorrosion und damit möglicherweise Ermüdungsbrüchen im Bereich der Konusverbindung reduziert wird [12, 13]. Das Verspannen der Implantatkomponenten mit dem TOV gemäß Instrumentationsanleitung [16] ist ein absolutes Muss, eine „sine qua non“, da eine alleinige Sicherung mit der Sicherungsschraube keine ausreichende Verbindungsfestigkeit gewährleisten kann – wie die in Folge der zyklischen Belastung größere Lockerung verglichen mit dem Anzugsmoment zeigte. Das Anziehen der Schraube ist empfindlich gegenüber axialer Verkippung des Drehmomentbegrenzers und des Knebels aufgrund von Reibung im Gewinde des Schaftes. Für die Testung wurden alle Schrauben mit ausgestreckten Armen auf der Materialprüfmaschine angezogen. Dieses Vorgehen lässt nicht vollständig ausschließen, dass das initial tatsächlich auf die Schraube aufgebrachte Torsionsmoment unterhalb von 25 Nm lag, trotz Auslösen des Drehmomentbegrenzers. Ein Einfluss auf die für die Verbindungsfestigkeit relevante Abzugskraft wurde jedoch nicht festgestellt.

In der klinischen Anwendung ist durch die signifikant höheren Relativbewegungen, insbesondere der Mikrobewegung und der Komponentenverdrehung, bei den verunreinigten Komponenten eine reduzierte Lebensdauer des Implantats zu erwarten, insbesondere bei fehlender Verspannung [2, 11, 12]. Die Notwendigkeit eines mehrmaligen Umgreifens am Knebel beim Anziehen der Sicherungsschraube ohne vorheriges Verspannen kann hier ein Hinweis auf eine mögliche Verunreinigung der Konusoberfläche sein, da Partikel das anfängliche Fügen von Hand erschweren.

## Fazit für die Praxis

- Mithilfe des torsionsfreien Vorspanninstruments (TOV) gefügte Implantate weisen signifikant höhere Abzugskräfte und eine geringere Relativbewegung und Komponentenverdrehung auf. Die Verwendung des TOV muss gewährleistet werden.
- Verunreinigung der Konusoberfläche führt zu signifikant höheren Relativbewegungen an der Konusverbindung, selbst bei korrekter Verspannung, und könnte somit mit einer erhöhten Korrosionsproblematik assoziiert sein.
- Notwendiges Umgreifen beim Anziehen der Sicherungsschraube unter Verwendung des Knebels deutet auf eine vorhandene Verunreinigung des Konus sowie ein nicht erfolgtes Verspannen hin.

## Abkürzungen

DIC: Digitale Bildkorrelation
TOV: Torsionsfreies Vorspanninstrument
